# Public auditing for real-time medical sensor data in cloud-assisted HealthIIoT system

**DOI:** 10.1007/s12200-022-00028-1

**Published:** 2022-06-29

**Authors:** Weiping Ye, Jia Wang, Hui Tian, Hanyu Quan

**Affiliations:** 1 College of Computer Science and Technology, Huaqiao University, Xiamen, 361021 China; 2 Wuhan National Laboratory for Optoelectronics, Wuhan, 430074 China; 3 Xiamen Key Laboratory of Data Security and Blockchain Technology, Xiamen, 361021 China; 4 Fujian Key Laboratory of Big Data Intelligence and Security, Xiamen, 361021 China

**Keywords:** Healthcare industrial internet of things (HealthIIoT), Medical sensor data, Online/offline signature, Public auditing

## Abstract

With the advancement of industrial internet of things (IIoT), wireless medical sensor networks (WMSNs) have been widely introduced in modern healthcare systems to collect real-time medical data from patients, which is known as HealthIIoT. Considering the limited computing and storage capabilities of lightweight HealthIIoT devices, it is necessary to upload these data to remote cloud servers for storage and maintenance. However, there are still some serious security issues within outsourcing medical sensor data to the cloud. One of the most significant challenges is how to ensure the integrity of these data, which is a prerequisite for providing precise medical diagnosis and treatment. To meet this challenge, we propose a novel and efficient public auditing scheme, which is suitable for cloud-assisted HealthIIoT system. Specifically, to address the contradiction between the high real-time requirement of medical sensor data and the limited computing power of HealthIIoT devices, a new online/offline tag generation algorithm is designed to improve preprocessing efficiency; to protect medical data privacy, a secure hash function is employed to blind the data proof. We formally prove the security of the presented scheme, and evaluate the performance through detailed experimental comparisons with the state-of-the-art ones. The results show that the presented scheme can greatly improve the efficiency of tag generation, while achieving better auditing performance than previous schemes.

**Graphical abstract:**

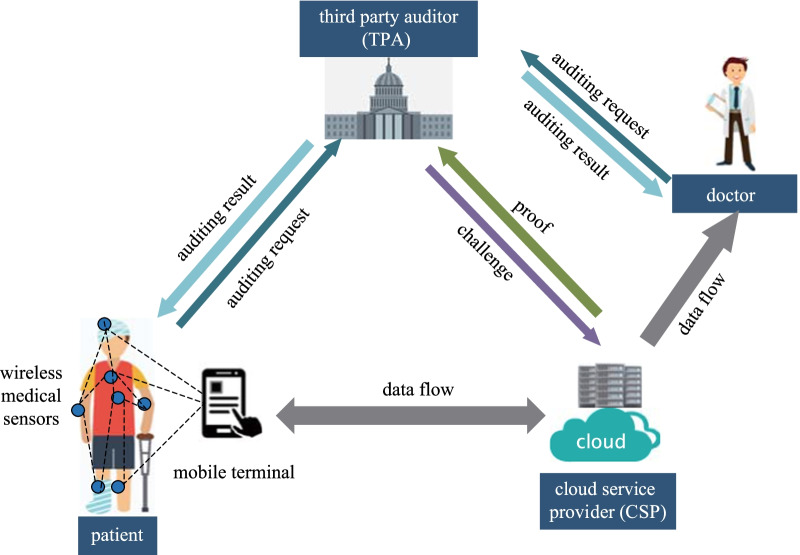

## Introduction

As a fast-growing application of internet of things (IoT) in the industrial sector, industrial IoT (IIoT), which can collect, monitor and deliver valuable information through embedded sensors, has shown enormous potential for improving quality of service in many industries [1, 2]. This is especially true in the field of healthcare. The promising IIoT has played a vital role in promoting the informatization and intelligence of healthcare systems, which is known as healthcare IIoT (HealthIIoT) [2, 3]. The HealthIIoT has been recognized as an important tool for providing real-time and high-quality healthcare services, where wireless medical sensors implanted inside or worn on patients are utilized for collecting health data, such as blood pressure, breathing pattern, heart rate, and so on [4–6]. These crucial health data can be remotely and flexibly accessed by doctors to diagnose the patients’ condition in time and conduct further treatment, thereby significantly improving the healthcare services [1–4]. As a key component of HealthIIoT, a wireless medical sensor network (WMSN) has been implemented to assist in containing the spread of COVID-19. Since early diagnosis and isolation are effective and imperative strategies for epidemic prevention and control, WMSN can effectively identity those who exhibit symptoms and are most likely to be infected with the virus, and help the diagnosis system to automatically collect COVID-19 data [7, 8]. HealthIIoT is thus profoundly changing the healthcare industry.

In HealthIIoT, it is important to support real-time operation and processing on a large amount of medical data collected by WMSN. Considering the limited computing and storage capabilities of the sensor devices, medical sensor data gleaned in WMSN are generally sent to the cloud for storage and maintenance [9–12]. With the almost infinite computing power and storage resources, cloud computing technology has been widely adopted in modern healthcare systems to provide infrastructures and services to make up for the technical limitations of HealthIIoT in communication, processing, and storage [10–12].

Under this circumstance, the cloud-assisted HealthIIoT system came into being [2, 4, 11, 12], in which WMSN collects vital health data from patients, such as physiologic parameters and motion data; and these medical sensor data are transmitted to patients’ mobile terminal for integration and preprocessing; then these preprocessed data will be immediately outsourced to remote cloud servers [2, 4, 11, 12]. Cloud-based medical data provides access anywhere/anytime, through which relevant doctors can deliver efficient, convenient and real-time healthcare services including health monitoring, disease prevention, diagnosis and treatment [12]. In brief, the cloud-assisted HealthIIoT paradigm enhances the ability of collection of vital health data in real-time, enables both patients and doctors to access information and interact in a cost-effective manner, and provides high-quality and efficient healthcare services [2, 4, 11, 12].

The cloud-assisted HealthIIoT system has many advantages, but it still faces some serious security and privacy challenges in practical application [2–4, 13]. One of the essentials is how to ensure the integrity of medical data outsourced to the cloud [14], which may be challenged by the following. First, after uploading medical data to remote cloud servers, the user loses substantial control over these data, which makes the traditional data security solution invalid in the cloud environment [15]. Second, for its own benefit, the cloud service provider (CSP) may conceal the data corruption fact caused by intentional and unintentional hardware and software failures [16]. In addition, frequent data loss accidents in recent years have seriously affected the trust relationship between the CSP and users [17]. Medical data are closely related to patients’ privacy and health maintenance. Once they are tampered with or partly lost, it will have a significant impact on medical diagnosis and treatment. Therefore, ensuring the integrity of medical sensor data in the cloud-assisted HealthIIoT system is an essential task.

To ensure the correctness and completeness of outsourced data, cloud auditing, also known as remote data integrity checking (RDIC), has emerged [18–20]. Generally speaking, there are two implementation models for cloud auditing, i.e., private auditing and public auditing. In the former, the verification operation is only performed between the user and CSP, which imposes a heavy computation and communication burden on user and may cause a dispute over the auditing result. To solve these problems, the public auditing model introduces a neutral third-party auditor (TPA) to perform the auditing process on behalf of the user, which can significantly reduce the user’s communication and computation costs, as well as provide a credible auditing result. Therefore, the public auditing model is believed to be the right direction of cloud auditing’s development [14, 16, 24–34].

With the continuous development of cloud computing, its application scenarios, security and efficiency requirements are becoming more and more diversified. To meet these challenges, a great number of auditing schemes have been proposed, such as dynamic data auditing [23–26], privacy-preserving auditing [27, 28], public auditing for the shared data [29–31], certificateless public auditing [32–34] and online/offline auditing [36, 37].

So far, cloud auditing has made great achievements. However, in the cloud-assisted HealthIIoT system, there are still some vital problems that have not been properly resolved.

The first is the contradiction between the high real-time requirement of medical sensor data and the limited computing power of HealthIIoT devices. Unlike general application scenarios, the HealthIIoT system has high real-time requirement for medical sensor data to provide continuous medical monitoring, timely health examination, prompt diagnosis and adequate treatment. However, most of existing cloud auditing schemes require users to perform expensive computations to preprocess the data before outsourcing them to the cloud, which undoubtedly places a heavy burden on lightweight devices, thereby making traditional auditing schemes inapplicable in the cloud-assisted HealthIIoT environment. Therefore, it is crucial to design an efficient tag generation algorithm for resource-constrained devices to preprocess medical sensor data in real time.

The second is the importance and sensitivity of medical sensor data. Public auditing introduces TPA to perform auditing process on behalf of the user, which can significantly reduce the user’s overheads, and provide a credible auditing result. However, there is a risk of data leakage, because TPA may obtain the sampled data content from the proof generated by the CSP during auditing process. Therefore, it is vital for public auditing scheme to protect data privacy from TPA.

To solve the problems mentioned above, this paper presents a novel public auditing scheme for cloud-assisted HealthIIoT system. Specifically, our major contributions in this work can be summarized as follows:

1. We propose an efficient public auditing scheme for cloud-based real-time medical sensor data, which is suitable for cloud-assisted HealthIIoT system that consist of a large number of lightweight devices.
2. To address the contradiction between the high real-time requirement of medical sensor data and the limited computing power of HealthIIoT devices, we design an efficient online/offline tag generation algorithm. Specifically, most of heavy computations are conducted offline while online tag generation only performs lightweight computations, thereby enabling resource-constrained devices to efficiently preprocess medical sensor data in real time before outsourcing them to the cloud.
3. To protect the privacy of medical sensor data, we employ a secure hash function to blind the data proof. According to the preimage resistance of the secure hash function, TPA cannot derive any actual data information from the data proof during the verification phase. Therefore, the presented scheme can take advantage of TPA to perform auditing process, while ensuring that TPA cannot directly or indirectly obtain any actual medical data information during the auditing process.
4. We formally prove the security of the presented scheme, and evaluate the performance by theoretical analyses and experimental comparisons with the state-of-the-art schemes. The results show that the presented scheme can efficiently achieve secure auditing for medical sensor data, and outperform previous schemes in terms of computation and communication costs.

The remainder of this paper is organized as follows. In Sect. 2, we review the related work. Section 3 introduces background and preliminaries. Then, we explain the presented scheme in detail in Sect. 4 and provide the security analysis in Sect. 5. Section 6 gives the performance evaluation of the presented scheme through theoretical analyses and experimental comparisons. Finally, we draw the conclusion of this work in Sect. 7.

## Related work

The cloud-assisted HealthIIoT system collects patients’ health data in real time through WMSN, and employs the cloud computing to store and manage these data, which provide patients and doctors with an open, flexible, and cost-effective platform. Despite these advantages, it still faces some serious security challenges. One of the biggest concerns is how to ensure the correctness and completeness of these cloud-based medical sensor data, because the intact and untampered medical data are a key prerequisite for providing accurate medical diagnosis and treatment.

Cloud auditing, which can effectively and securely verify whether the CSP is honestly and correctly storing the outsourced data, has received extensive attention from both academia and industry. There are two kinds of implementation models for cloud auditing, namely, the private auditing and the public auditing. As one of the earliest private auditing schemes, Juels et al. [21] presented proof of retrievability (PoR) to ensure the data possession in the cloud. In the private auditing, the auditing task is directly performed between the CSP and user, which definitely increases the computation and communication burden on user and makes the auditing result controversial. To address these issues, Ateniese et al. [22] first proposed a public auditing scheme, i.e., provable data possession (PDP), which allows a TPA to implement the auditing on behalf of the user. The PDP scheme achieves public auditing, which greatly reduces the burden on user while providing a more reliable and dependable verification result. Subsequently, a great number of successful cloud auditing schemes have been proposed to meet various novel and distinctive requirements for cloud storage services.

For supporting dynamic data, some auditing schemes introduced different kinds of authenticated data structures to ensure data freshness in addition to verifying data integrity. Erway et al. [23] first introduced the rank-based authenticated skip list to present the dynamic PDP, which sets a general auditing framework for dynamic data. Wang et al. [24] employed the merkle hash tree (MHT) to achieve public auditing for dynamic data, in which the root value of MHT is generated as the verification proof to ensure the latest version. Zhu et al. [25] designed the index-hash table (IHT), which is stored in the TPA instead of CSP, to reduce the computation and communication costs. However, the sequential structure of the IHT is not applicable for update operation such as inserts and deletes. Subsequently, Tian et al. [26] presented a 2-dimension authenticated data structures called dynamic hash table (DHT) to achieve efficient updating performance.

To prevent the TPA from extracting the data content through linear proof combinations, a number of auditing schemes [27, 28] adopted random masking to blind data proof to protect data privacy, which is mainly divided into two kinds of strategies. In the first one [27], the CSP generates a mask number *R* = *yr* to blind the data proof* M* by computing *Mʹ* = *M* + *rH*(*R*), in which *y* is a global parameter, *r* is a number chosen randomly, and *H* is a hash function. In the other one [28], the TPA first computes a mask number *R* = *y*^*r*^ with a random number *r* and a global parameter *y*, then transmits *R* to CSP together with the challenge message; the CSP generates the masked data proof of *M* as *Mʹ* = *e*(*u*, *R*)^*M*^ to respond to the challenge, where *e* is a bilinear map and *u* is a global parameter.

With the increasing popularity of cloud collaboration, some auditing schemes for shared data, which can be accessed and processed by various users in a group, have been proposed. In addition to checking data integrity, shared data auditing should further support privacy preservation [29], identity traceability [30], and group dynamics [31]. For example, Wang et al. [29] proposed a public auditing scheme for shared data called Oruta, which uses the ring signature to generate verifiable tags to protect the user’s identity privacy. Yang et al. [30] designed an identity-block list (IBL) to record modification information of all data blocks, which can achieve the traceability of data modification. Tian et al. [31] designed a novel lazy-revocation mechanism to ingeniously achieve dynamic management of user groups.

To address certificate management in the traditional public key cryptography and key escrow in identity-based cryptography, Wang et al. [32] first introduced the certificateless signature into a cloud auditing scheme, in which the user’s private key included two independent parts that are generated by semi-trusted key generation center and user respectively. He et al. [33] proposed a certificateless public auditing scheme for cloud-assisted wireless body area networks. For the group sharing data under multiple users, Li et al. [34] designed a corresponding certificateless solution to address the user revocation issue.

To achieve efficient cloud auditing for resource-constrained devices, the online/offline signature, which was first proposed by Even et al. [35], has been introduced into PDP schemes, where the verifiable tags are generated in two phases: the offline phase and the online phase. That is, the heavy computations for generating verifiable tags can be performed offline in advance, thereby achieving an efficient online tag generation in real time. Li et al. [36] proposed two privacy-preserving public auditing protocols for lightweight devices using online/offline signatures: the basic one and the improved one. Specifically, the basic protocol is only practical for short data. The improved protocol utilizes the MHT to eliminate this restriction and support the auditing of dynamic data, but the authenticated data structure would incur heavy computation and communication costs. Wang et al. [37] presented a semi-generic online/offline PDP trans-formation framework which is applicable to PDP-related schemes with metadata aggregate ability and public metadata expansibility. However, such a general auditing model was not optimized for specific application scenarios.

Although a great number of successful auditing schemes have been proposed to meet various requirements, they cannot be directly applied in cloud-assisted HealthIIoT system, because of the particularities of medical sensor data, such as the high real-time requirement and the sensitivity of medical data. Thus, in this paper, we are motivated to present a tailored efficient public auditing scheme for medical sensor data in the cloud.

## Background and preliminaries

### System model

**Fig. 1:**
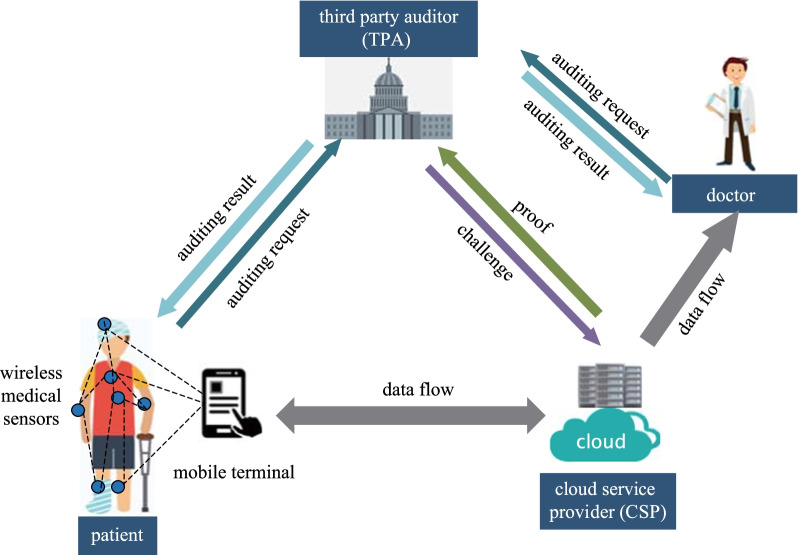
Public auditing model for medical sensor data in the cloud-assisted HealthIIoT system

The system model of the presented scheme is shown in Fig. 1, which includes four types of entities, i.e., CSP, Patient (including wireless medical sensors and mobile terminal), Doctor, and TPA.

#### Cloud service provider (CSP)

An entity with powerful computing resources and storage spaces, providing scalable and on-demand data storage and maintenance services.

#### Patient

The data owner, includes wireless medical sensors and mobile terminal. *Wireless medical sensors* continuously collect the patient’s health data in real time, and periodically transmit medical sensor data to *mobile terminal* that integrates and preprocesses these data. Finally, the patient uploads these preprocessed data to the CSP through the mobile terminal. The patient authorizes TPA to check the correctness and completeness of outsourced data in the cloud.

#### Doctor

The data user, can access the real-time medical sensor data stored in the cloud. Before utilizing medical cloud data to perform diagnosis and treatment, the doctor authorizes TPA to verify data integrity.

#### Third party auditor (TPA)

A neutral entity, is authorized to verify the integrity of medical sensor data stored in the cloud on behalf of the patient and doctor.

In the cloud-assisted HealthIIoT system, wireless medical sensor network collects patient’s health data and transmits them to a mobile terminal (such as smart phone and smart watch). This lightweight mobile terminal performs the preprocessing of these data, which includes dividing the data into multiple blocks and generating a corresponding tag for each data block. Finally, these preprocessed data are outsourced to remote cloud servers.

Patients enjoy storage and maintenance services by outsourcing medical data to the CSP. However, since patients have lost the substantial control over these data, they may be keen to check the correctness and integrity of their data periodically by authorizing the TPA to perform the auditing process. Meanwhile, before further analyzing cloud-based medical data, doctors need to ensure the data integrity to prevent the data from being tampered with or partially deleted, which will affect the diagnosis and even lead to misdiagnosis.

### Threat model

Usually, the CSP would provide a dependable storage service as requested, but the CSP may cover up the data corruption fact to maintain its own credibility and business interest. In addition, the TPA is assumed to be credible but curious. Specifically, the TPA can perform auditing tasks according to users’ requirements, but it may be curious about the content of medical data. The CSP may launch the following attacks to pass the verification performed by the TPA:

- Forging attack: the CSP tries to forge the data blocks and corresponding tags to pass the verification.
- Replacing attack: the CSP attempts to use other data blocks and corresponding tags that are stored well in the cloud as a replacement for damaged data blocks and tags.
- Replaying attack: the CSP tries to use the proof information generated in the previous auditing process to deceive the TPA.

### Design goals

In the presented scheme, we try to achieve the following objectives to effectively support public auditing for real-time medical sensor data in cloud-assisted HealthIIoT system under the above threats:

1. Public auditing: Any TPA authorized by users can verify the integrity of medical sensor data in the cloud.
2. Blockless verification: The TPA does not need to retrieve the whole file to check data integrity.
3. Storage correctness: The CSP that does not correctly store patients’ data as required cannot pass the verification.
4. Lightweight: The auditing process should be performed with the minimum communication and computation costs.
5. Data privacy preservation: The TPA is assumed to be credible but curious, so it is necessary to ensure that the TPA cannot directly or indirectly obtain any actual data information during the auditing process.
6. Batch auditing: the TPA can perform multiple auditing tasks from different patients and doctors simultaneously in a cost-effective manner.

### Preliminaries

#### Bilinear map

Let ${\mathbb{G}}$_1_ and ${\mathbb{G}}$_2_ be two multiplicative cyclic groups with the large prime order *p*, and *g* be the generator of ${\mathbb{G}}$_1_. A bilinear map *e*: ${\mathbb{G}}$_1_ × ${\mathbb{G}}$_1_ → ${\mathbb{G}}$_2_ has the following properties:

1. Computability: The bilinear map *e* should be efficiently computable.
2. Bilinearity: For ∀ *x*, *y*, *l* ∈ ${\mathbb{G}}$_1_ and ∀ *a*, *b* ∈ ℤ_*p*_, *e*(*x*^*a*^, *y*^*b*^) = *e*(*x*, *y*)^*ab*^, and *e*(*x*, *y*·*l*) = *e*(*x*·*l*, *y*) = *e*(*x*, *y*)·*e*(*x*, *l*).
3. Non-degeneracy: *e*(*g*, *g*) ≠ 1.

#### Zhang–Safavi–Susilo signature (ZSS signature)

Based on the bilinear pairings, Zhang et al. [38] proposed an efficient short signature scheme, which requires fewer pairing operations and is more efficient than a Boneh–Lynn–Shacham (BLS) signature [39]. Let ${\mathbb{G}}$_1_ and ${\mathbb{G}}$_2_ be the multiplicative cyclic groups of a large prime order *q*, where *P* is a generator of ${\mathbb{G}}$_1_. *H*_1_ is a secure hash function with *H*_1_: {0, 1}* → *ℤ*_p_*, and *e*: ${\mathbb{G}}$_1_ × ${\mathbb{G}}$_1_ → ${\mathbb{G}}$_2_ is a bilinear map. ZSS signature consists of the following four algorithms: a parameter generation algorithm *ParamGen*, a key generation algorithm *KeyGen*, a signature generation algorithm *Sign* and a signature verification algorithm *Ver*.

1. *ParamGen*. The system parameters are {${\mathbb{G}}$_1_, ${\mathbb{G}}$_2_, *e*, *q*, *P*, *H*}.
2. *KeyGen*. Randomly selects *x* ∈ *ℤ*_p_* as the secret key, and computes the public key as *P*_pub_ = *xP*.
3. *Sign*. Given a secret key *x*, and a message *m*, computes the signature *S* as follow: $S = \frac{1}{H(m) + x}P.$
4. *Ver*. Given a public key *P*_pub_, a message *m*, and a signature *S*, verify if $e(H(m)P + P_{\text{pub}} ,S) = e(P,P).$

#### Computational Diffie–Hellman (CDH) assumption

Let ${\mathbb{G}}$ be a multiplicative cyclic group with the large prime order *q*, and *P* be the generator of ${\mathbb{G}}$. For unknown *a*, *b* ∈ *ℤ*_p_*, given *P*, *aP* and *bP*, it is computationally infeasible to compute *abP*.

#### Discrete logarithm (DL) assumption

Let ${\mathbb{G}}$ be a multiplicative cyclic group of a large prime order *q*. Given two group elements *P* and *Q*, it is computationally infeasible to find an integer *n* ∈ *ℤ*_p_* where *Q* = *nP*.

## Presented scheme

In the cloud-assisted HealthIIoT system, wireless medical sensors implanted inside or on the patient continuously collect important health data, and periodically transmit medical sensor data to the patient’s lightweight mobile terminal such as smart phone and smart watch. After integrating and preprocessing these data, the patient uploads the real-time data and corresponding verifiable tags to remote cloud servers for storage and maintenance. Upon receiving these data, the CSP will analyze and process the medical data in real time. In the meantime, relevant doctors can remotely access the processed medical sensor data to monitor the patient’s health status, conduct detailed analysis and perform a quick and efficient diagnosis and treatment.

The correct and complete medical cloud data are a key prerequisite for providing precise medical diagnosis, treatment, and further analysis. Therefore, patients and relevant doctors desire to ensure the correctness and completeness of medical data in the cloud. However, most existing public auditing schemes require users to conduct expensive computations, which is not suitable for lightweight devices. To address the contradiction between the real-time requirement of medical sensor data and the resource-constrained wireless sensor devices and mobile terminal in HealthIIoT, we introduce the online/offline signature mechanism into the public auditing for real-time medical sensor data in the cloud. Specifically, the data tag generation is divided into two phases, that is, phase 1: offline tag generation and phase 2: online tag generation. The offline tag generation is performed by the mobile terminal before it receives medical sensor data and in which most heavy computations are executed. The offline tag generation can be conducted when the mobile terminal is idle or in the middle of a transmission gap from medical sensors. Upon receiving medical sensor data, the mobile terminal only needs to perform lightweight computation with the offline pre-computed results, where the online tag generation can be executed very efficiently. Therefore, the online/offline tag generation mechanism is very applicable for the HealthIIoT system, which greatly reduces the computation burden on the lightweight mobile terminal and meets the high real-time requirement of medical sensor data.

The presented scheme consists of four polynomial-time procedures, i.e., System initialization, Key generation, Tag generation (offline and online phases), and Auditing. Table 1 lists some notations to be used in the presented scheme.

**Table 1:** Notations

Notation	Description	Notation	Description
*E*(*F*_*l*_)	An elliptic curve on the finite field *F*_*l*_	*q*	A large prime order
${\mathbb{G}}$_1_, ${\mathbb{G}}$_2_	Two multiplicative cyclic groups	*P*	A generator of ${\mathbb{G}}$_1_
*H*_1_	A hash function: {0, 1}* → *ℤ*_p_*	*e*: ${\mathbb{G}}$_1_ × ${\mathbb{G}}$_1_ → ${\mathbb{G}}$_2_	A bilinear map
*H*_2_	A hash function: ${\mathbb{G}}$_1_ → *ℤ*_p_*	(*Y* = *xP*, *x*)	The trapdoor hash function key pair
*sk* = *a*	The user’ private key	*pk* = *aP*	The user’s public key
(*w*_*i*_, *r*_*i*_)	A random number *w*_*i*_ and auxiliary parameter *r*_*i*_	*fid*	The file identifier
*σ*_*i*_	The offline block tag	*F* = {*m*_1_, *m*_2_, …, *m*_*n*_}	*m*_*i*_ is the data block of the file *F*
*r*_*i*_*ʹ*	The online block tag	*chal* = {*i*, *v*_*i*_|*i* ∈ *L*}	The challenge information
Ω	The data proof	T	The tag proof

### System initialization

Let *l* be a prime power, and *E*(*F*_*l*_) be an elliptic curve on the finite field *F*_*l*_. *P* is a point on *E*(*F*_*l*_) of a large prime order *q*. Let ${\mathbb{G}}$_1_ and ${\mathbb{G}}$_2_ be the multiplicative cyclic groups, where *P* is a generator of ${\mathbb{G}}$_1_. *H*_1_ and *H*_2_ are two secure hash functions with *H*_1_: {0, 1}* → *ℤ*_p_*, *H*_2_: ${\mathbb{G}}$_1_ → *ℤ*_p_*, and *e*: ${\mathbb{G}}$_1_ × ${\mathbb{G}}$_1_ → ${\mathbb{G}}$_2_ is a bilinear map. Finally, the system parameters are set as *SP* = {*E*, *l*, *q*, *P*, ${\mathbb{G}}$_1_, ${\mathbb{G}}$_2_, *H*_1_, *H*_2_}.

### Key generation

The user first selects a random number *x* ∈ *ℤ*_p_* to generate the trapdoor hash function key pair (*Y* = *xP*, *x*). Then, *a* ∈ *ℤ*_p_* is randomly chosen as the user’s private key *sk*, i.e., *sk* = *a*, and the public key is *pk* = *aP*.

### Tag generation

The tag generation procedure is mainly composed of the following two phases: offline tag generation and online tag generation.

#### Phase 1 (Offline tag generation)

Without receiving actual medical sensor data from WMSN, the patient randomly generates a series of random numbers *w*_*i*_ and auxiliary parameters *r*_*i*_, where (*w*_*i*_, *r*_*i*_) ∈ *ℤ*_p_* and 1 ≤ *i* ≤ *k*. Meanwhile, (*w*_*i*_, *r*_*i*_) are mapped to an element *h*_*i*_ of the group ${\mathbb{G}}$_1_ through a chameleon hash function with the trapdoor key as follow:

1 $$h_{i} = w_{i} P + r_{i} Y.$$

A random number *fid* ∈ *ℤ*_p_* is chosen as the file identifier. Furthermore, the patient generates the offline block tag *σ*_*i*_ using his/her private key *sk* as follow:

2 $$\sigma_{i} = \frac{1}{{H_{1} (fid||i) + H_{2} (h_{i} )a}}P.$$

Finally, the patient gets the offline block tag pool {*σ*_*i*_}_1 ≤ *i* ≤ *k*_ that will be used in the online phase, where *k* is the maximum number of data blocks.

#### Phase 2 (Online tag generation)

Upon receiving the medical data file *F*, mobile terminal of the patient first divides the file *F* ∈ {0, 1}* into *n* data blocks, namely *F* = {*m*_1_, *m*_2_, …, *m*_*n*_}, where *m*_*i*_ is the data block, *i* ∈ [1,*n*] and *n* ≤ *k*. For each data block *m*_*i*_ (1 ≤ *i* ≤ *n*), the online block tag *r*_*i*_*ʹ* is calculated as follow:

3 $$r^{\prime}_{i} = x^{ - 1} (w_{i} - m_{i} ) + r_{i} \bmod q.$$

So far, the patient generates the complete data block tag {*σ*_*i*_, *r*_*i*_*ʹ*}_1 ≤ *i* ≤ *n*_. Finally, the preprocessed file {*fid*, *F*, {*σ*_*i*_, *r*_*i*_*ʹ*}_1 ≤ *i* ≤ *n*_} is uploaded to CSP. It is worth noting that *x*^−1^ can be calculated in the offline phase to further reduce the online computation burden.

Upon the receipt of these medical sensor data and their corresponding block tags, the CSP first calculates the chameleon hash as follow:

4 $$h_{i}^{\prime } = m_{i} P + r^{\prime}_{i} \cdot Y.$$

It then checks the validity of block tags as follow:

5 $$e(H_{1} (fid{||}i) \cdot P + H_{2} (h^{\prime}_{i} ) \cdot pk,\sigma_{i} ) = e(P,P).$$

If the equation holds, the CSP accepts, and the patient can delete them locally; otherwise, the CSP rejects their storage and requests the patient to re-upload the correct medical data and tags.

### Auditing

When patients want to check whether their medical data in the cloud are completely and correctly stored, or relevant doctors need to utilize medical sensor data to analyze patients’ health condition, they need to ensure the correctness and completeness of medical data in the cloud. The auditing process in the presented scheme includes the following three steps:

- **Step 1 (Challenge)**: To check the integrity of cloud-based medical sensor data, patients or the appropriate doctors make an auditing request to the TPA. Upon receiving the auditing request, the TPA randomly selects *c* data blocks from all data blocks to form a challenge set *L* = {*l*_1_, *l*_2_, …, *l*_*c*_ | 1 ≤ *l*_*i*_ ≤ *n*} and chooses a random number *v*_*i*_ ∈ *ℤ*_p_* for each *i* ∈ *L*. Finally, the TPA sends the challenge *chal* = {*i*, *v*_*i*_ | *i* ∈ *L*} to CSP.
- **Step 2 (Proof generation)**: Upon receiving the challenge information, the CSP first calculates the chameleon hash value as follow:
  6 $$h_{i}^{\prime } = m_{i} P + r^{\prime}_{i} \cdot Y.$$
  Then, the data proof Ω and tag proof T are calculated as follows:
  7 $$\Omega = \sum\limits_{i \in L} {v_{i} H_{2} (h^{\prime}_{i} } ),$$
  8 $${\rm T} = P - P^{2} \sum\limits_{i \in L} {\frac{{v_{i} }}{{\sigma_{i} }}} .$$
  In the generation of data proof, we take advantage of the preimage resistance of the secure hash function to protect data privacy in the following verification. Finally, the CSP returns the proof *pf* = {Ω, T} to the TPA.
- **Step 3 (verification)**: Upon the receipt of the proof *pf* = {Ω, T} from the CSP, the TPA checks the data integrity with the following equation:
  9 $$e({\text{T}},P) \cdot e \bigg( \Omega \cdot pk + \sum\limits_{i \in L} {H_{1} (fid||i) \cdot v_{i} } \cdot P,P \bigg) = e(P,P).$$
  If the equation holds, it outputs TRUE; otherwise, it outputs FALSE.

## Security analysis

In this section, some security analyses concerning the proposed scheme will be presented, including correctness, secure signature, collision resistance of the chameleon hash function, unforgeability of the proof and data privacy preservation.

### **Theorem 1 (Correctness)**

*If the CSP is honestly and correctly storing the outsourced data, then the response proof generated by CSP can pass the verification challenged by TPA.*

### *Proof*

According to the characteristics of the bilinear map, Eq. (9) in the verification phase of the auditing process can be proven correct as follow:

$$\begin{aligned} &e({\text{T}},P) \cdot e\left(\Omega \cdot pk + P\sum\limits_{i \in L} {H_{1} (fid||i)v_{i} } ,P\right) \hfill \\ &= e\left(P - P^{2} \sum\limits_{i \in L} {\frac{{v_{i} }}{{\sigma_{i} }}} ,P\right) \cdot e\left(\sum\limits_{i \in L} {(v_{i} H_{2} (h_{i} ))} \cdot aP + P\sum\limits_{i \in L} {H_{1} (fid||i)v_{i} } ,P\right) \hfill \\ &= e\left(P - P^{2} \sum\limits_{i \in L} {v_{i} (H_{1} (fid||i) + H_{2} (h_{i} )a)\frac{1}{P}} ,P\right) \cdot e\left(P\sum\limits_{i \in L} {v_{i} (H_{2} (h_{i} } )a + H_{1} (fid||i)),P\right) \hfill \\ &= e\left(P \bigg(1 - \sum\limits_{i \in L} {v_{i} (H_{1} (fid||i) + H_{2} (h_{i} )a)\bigg)} ,P\right) \cdot e\left(P\sum\limits_{i \in L} {v_{i} (H_{2} (h_{i} } )a + H_{1} (fid||i)),P\right) \hfill \\ &= e(P,P). \hfill \\ \end{aligned}$$

### Theorem 2 (Secure signature)

*The signature scheme S = <System initialization, Key generation, Tag generation, Auditing> in this work is designed based on ZSS signature *[38]*, which is infeasible for a forger who only knows the public key to produce a valid block-signature pair after obtaining polynomially many signatures on data blocks.*

### *Proof*

This theorem follows from ZSS signature [38], where it has been proven it is existentially unforgeable under an adaptive chosen message attack with the assumption that the CDH problem is hard in bilinear groups. The proof can be found in Ref. [38], and is omitted here.

### **Theorem 3 (Collision resistance)**

*The chameleon hash function in the presented scheme is collision resistant under the DL assumption.*

### *Proof*

Suppose there is a probabilistic polynomial-time algorithm *ε* which on input a public hash key *Y*, outputs two distinct pairs of data block and auxiliary parameter (*m*_*i*_*, r*_*i*_) and (*m*_*i*_*ʹ*, *r*_*i*_*ʹ*) such that *m*_*i*_ ≠ *m*_*i*_*ʹ* and *m*_*i*_·*P* + *r*_*i*_·*Y* = *m*_*i*_*ʹ*·*P* + *r*_*i*_*ʹ*·*Y*. However, it contradicts the DL assumption, where it is computationally infeasible to compute *Y* = *xP*. Therefore, the chameleon hash function in the presented scheme is collision resistant.

### **Theorem 4 (Unforgeability of the proof)**

*In the presented scheme, it is computationally infeasible for the CSP to forge a valid proof to pass the verification. That is, the presented scheme can effectively resist the forging attack from the CSP.*

### *Proof*

Upon receiving the challenge *chal* = {*i*, *v*_*i*_|*i* ∈ *L*} from the TPA, the CSP generates the corresponding proof *pf* = {Ω, T} that responds to the challenge. We prove the unforgeability of Ω and T respectively as follows:

1. Unforgeability of Ω

The following game is designed to prove the unforgeability of Ω: the CSP provides forged proof information *pf*′ = {Ω′, T} to respond to the challenge from the TPA, where

10 $$\Omega = \sum\limits_{i \in L} {v_{i} H_{2} (h_{i}^{\prime } } ) \ne \Omega^{\prime} = \sum\limits_{i \in L} {v_{i} H_{2} (h_{i}^{\prime \prime } } ).$$

As Eq. (10) suggests, ∃*i* ∈ *L*, $h^{\prime}_{i} \ne h^{\prime\prime}_{i}$. If the CSP passes the verification with forged proof information *pf′* = {Ω′, T}, the CSP wins the game; otherwise, the CSP fails.

Assume that the CSP wins the game, then

11 $$\begin{aligned} &e({\text{T}},P) \cdot e\left(\Omega^{\prime} \cdot pk + \sum\limits_{i \in L} {H_{1} (fid||i) \cdot v_{i} } \cdot P,P\right) \hfill \\ &= e({\text{T}},P) \cdot e\left(\sum\limits_{i \in L} {v_{i} H_{2} (h^{\prime\prime}_{i} } ) \cdot pk + \sum\limits_{i \in L} {H_{1} (fid||i) \cdot v_{i} } \cdot P,P\right) \hfill \\ &= e(P,P). \hfill \\ \end{aligned}$$

The correct proof information is *pf* = {Ω, T}, so we can get

12 $$\begin{aligned} &e({\text{T}},P) \cdot e\left(\Omega^{\prime} \cdot pk + \sum\limits_{i \in L} {H_{1} (fid||i) \cdot v_{i} } \cdot P,P\right) \hfill \\ &= e({\text{T}},P) \cdot e\left(\sum\limits_{i \in L} {v_{i} H_{2} (h_{i}^{\prime} } ) \cdot pk + \sum\limits_{i \in L} {H_{1} (fid||i) \cdot v_{i} } \cdot P,P\right) \hfill \\& = e(P,P). \hfill \\ \end{aligned}$$

According to the bilinear mapping described in Sect. 3, we can deduce that $h^{\prime}_{i} = h^{\prime\prime}_{i}$, ∀*i* ∈ *L*, which contradicts the assumption. Therefore, we can conclude that Ω cannot be forged.

(2) Unforgeability of T

We design the following game to prove the unforgeability of T: the CSP provides a forged proof *pfʺ* = {Ω, Tʹ} to respond to the challenge, where

13 $${\text{T}} = P - P^{2} \sum\limits_{i \in L} {\frac{{v_{i} }}{{\sigma_{i} }}} \ne {\rm T}^{\prime} = P - P^{2} \sum\limits_{i \in L} {\frac{{v_{i} }}{{\sigma^{\prime}_{i} }}} .$$

As Eq. (13) suggests, ∃*i* ∈ *L*, ${\sigma }_{i}\ne {\sigma }_{i}^{^{\prime}}$. If the CSP passes the verification with the forged proof *pfʺ* = {Ω, Tʹ}, the CSP wins the game; otherwise, the CSP fails.

Assume that the CSP wins the game, then,

14 $$e\left(P - P^{2} \sum\limits_{i \in L} {\frac{{v_{i} }}{{\sigma^{\prime}_{i} }}} ,P\right) \cdot e\left(\Omega \cdot pk + \sum\limits_{i \in L} {H_{1} (fid||i) \cdot v_{i} } \cdot P,P\right) = e(P,P).$$

Based on the correct proof *pf* = {Ω, T}, we have

15 $$e\left(P - P^{2} \sum\limits_{i \in L} {\frac{{v_{i} }}{{\sigma_{i} }}} ,P\right) \cdot e\left(\Omega \cdot pk + \sum\limits_{i \in L} {H_{1} (fid||i) \cdot v_{i} } \cdot P,P\right) = e(P,P).$$

According to the bilinear mapping, we can deduce that ${\sigma }_{i}={\sigma }_{i}^{^{\prime}}$, ∀*i* ∈ *L*, which contradicts the assumption. Therefore, we can conclude that T cannot be forged.

In summary, the presented scheme can effectively resist the forging attack.

### **Theorem 5 (Data privacy preservation)**

*In the presented scheme, the TPA cannot obtain the specific content of any medical sensor data from the proofs received from the CSP. That is, the TPA is only authorized to verify data integrity and should not learn any actual data information during the auditing process.*

### *Proof*

In the proof generation phase of the auditing process, the CSP generates the following proof information *pf* = {Ω, T}, where Ω is the data proof and T is the tag proof.

16 $$\Omega = \sum\limits_{i \in L} {v_{i} H_{2} (h^{\prime}_{i} } ),$$

17 $${\rm T} = P - P^{2} \sum\limits_{i \in L} {\frac{{v_{i} }}{{\sigma_{i} }}} .$$

Then the CSP returns proof *pf* = {Ω, T} to the TPA.

We can know that T is the aggregate value of the block tags *σ*_*i*_, which does not contain any content of medical data. Although Ω is the data proof that aggregated by medical data *m*_*i*_, in which *h*ʹ_*i*_ = *m*_*i*_·*P* + *r*ʹ_*i*_·*Y*. According to the preimage resistance of the secure hash function, the TPA cannot derive any actual data information *m*_*i*_ from Ω during the verification phase.

Therefore, the presented scheme can protect the privacy of medical sensor data from the TPA during auditing process.

## Performance evaluation

In this section, we make theoretical analyses and evaluate the performance by detailed experiments and comparisons with the state-of-the-art schemes [33, 37].

### Theoretical analyses

#### Communication costs

We compare communication costs during the auditing process among the presented scheme (called PAMSD) and state-of-the-art ones (i.e., CLPA [33], OOPDP [37]), which are summarized in Table 2. In the challenge phase, the communication costs of three schemes are the same; all are *c*·(|${\mathbb{Z}}_{p}^{*}$| + |*N*|). By contrast, in the proof generation phase, the communication costs of OOPDP are related to the number of segments in data block, which are |${\mathbb{G}}$_1_| + (*s* + 1)·|${\mathbb{Z}}_{p}^{*}$|. Therefore, the communication costs of OOPDP are much higher than those of CLPA and PAMSD. Moreover, the communication costs of CLPA and PAMSD are both |${\mathbb{G}}$_1_| + |${\mathbb{Z}}_{p}^{*}$|, but PAMSD employs a secure hash function to blind the data proof to support data privacy preservation in the proof generation, which is crucial to medical data.

**Table 2:** Comparison of communication costs

Schemes	Challenge	Proof generation
CLPA [33]	*c*·(|${\mathbb{Z}}_{p}^{*}$| + |*N*|)	|${\mathbb{G}}$_1_| + |${\mathbb{Z}}_{p}^{*}$|
OOPDP [37]	*c*·(|${\mathbb{Z}}_{p}^{*}$| + |*N*|)	|${\mathbb{G}}$_1_| + (*s* + 1)·|${\mathbb{Z}}_{p}^{*}$|
PAMSD	*c*·(|${\mathbb{Z}}_{p}^{*}$| + |*N*|)	|${\mathbb{G}}$_1_| + |${\mathbb{Z}}_{p}^{*}$|

*c* is the number of challenged blocks; *s* is the number of segments in the data block; |*N*| is the size of the elements in the set [1, *n*]; |${\mathbb{Z}}_{p}^{*}$| is the size of the elements in the group ${\mathbb{Z}}_{p}^{*}$; |${\mathbb{G}}$_1_| is the size of the elements in the group ${\mathbb{G}}$_1_

In summary, PAMSD is superior to CLPA and OOPDP in terms of communication costs and data privacy preservation.

#### Computation costs

Table 3 respectively lists the computation costs of the presented scheme and the two comparison schemes in the offline tag generation, online tag generation, proof generation, and verification phases. CLPA does not support online/offline tag generation mechanism, so it can be considered that all tags generation in CLPA are performed online. Therefore, in the offline tag generation phase, we only compare the computation costs of PAMSD with those of OOPDP. As shown in Table 3, the computation costs of PAMSD in the offline tag generation are *n*·(*Hash*_*G*1_ + 3*Mul*_*G*1_ + *Invert*_*Zp*_ + *Hash*_*Zp*_), which are slightly higher than 2*n*·*Exp*_*G*1_ of OOPDP. However, in the online tag generation phase, the computation costs of PAMSD are only *n*·*Mul*_*Zp*_, which are much lower than those of either CLPA or OOPDP. Considering the high real-time requirement of medical sensor data, which is critical for the cloud-assisted HealthIIoT system, it is appropriate to exchange slightly higher offline computation costs for online efficiency. It is worth noting that CLPA had the highest computation costs among three schemes, because it performed the entire tag generation online. Therefore, online/offline tag generation mechanism can greatly improve online efficiency.

**Table 3:** Comparison of computation costs

Schemes	CLPA [33]	OOPDP [37]	PAMSD
Offline tag generation	–	2*n*·*Exp*_*G*1_	*n*·(*Hash*_*G*1_ + 3*Mul*_*G*1_ + *Invert*_*Zp*_ + *Hash*_*Zp*_)
Online tag generation	(*n* + 1)·*Hash*_*G*1_ + 2*n*·*Mul*_*G*1_ + *n*·*Add*_*G*1_	*n*·(*s* + 2)·*Mul*_*Zp*_ + *n*·*s*·*Exp*_*Zp*_ + *n*·*Hash*_*Zp*_	*n*·*Mul*_*Zp*_
Proof generation	*c*·*Mul*_*G*1_ + (*c* − 1)·*Add*_*G*1_	(*c* + *s* − 2)·*Mul*_*G*1_ + (*c* + 1)·*Exp*_*G*1_	(3*c* + 1)·*Mul*_*G*1_ + *c*·(*Invert*_*G*1_ + *Hash*_*G*1_) + *c*·*Add*_*G*1_
Verification	2*Pair* + (*c* + 3)·*Mul*_*G*1_ + (*c* + 2)·*Add*_*G*1_ + (*c* + 2)·*Hash*_*G*1_ + 2*Hash*_*Zp*_	3*Pair* + *Invert*_*G*1_ + 2*Mul*_*G*1_ + 3*Exp*_*G*1_ + *c*·*Hash*_*Zp*_ + *Mul*_*G*2_	2*Pair* + *Add*_*G*1_ + *c*·*Hash*_*Zp*_ + 2*Mul*_*G*1_ + *Mul*_*G*2_

*c* is the number of challenged blocks; *n* is the number of data blocks; *s* is the number of segments in the data block; *Hash*_*Zp*_ is the execution time of the hash function *H*_1_: {0, 1}* → ${\mathbb{Z}}_{p}^{*}$; *Hash*_*G*1_ is the execution time of the hash function *H*_2_: ${\mathbb{G}}$_1_ → ℤ_p_*; *Exp*_*G*1_ and *Exp*_*Zp*_ are the average time of exponential operation of group ${\mathbb{G}}$_1_ and ℤ_p_* respectively; *Mul*_*G*1_ and *Mul*_*G*2_ are the average time of multiplication operations on group ${\mathbb{G}}$_1_ and ${\mathbb{G}}$_2_ respectively; *Pair* is the average time to perform the pairing operation; *Invert*_*G*1_ and *Invert*_*Zp*_ are the time for the inversion operation on group ${\mathbb{G}}$_1_ and ℤ_p_*; *Add*_*G*1_ is the average time of the addition operation on the group ${\mathbb{G}}$_1_

In the proof generation phase, the computation costs of PAMSD are (3*c* + 1) ·*Mul*_*G*1_ + *c*·(*Invert*_*G*1_ + *Hash*_*G*1_) + *c*·*Add*_*G*1_, which are slightly higher than those of either CLPA or OOPDP. However, the proof is generated by the CSP with significant computing power, which will not affect the overall performance of the auditing process. In fact, cloud servers are supposed to do the heavy computations for users. In the verification phase, the computation costs of PAMSD are 2*Pair* + *Add*_*G*1_ + *c*·*Hash*_*Zp*_ + 2*Mul*_*G*1_ + *Mul*_*G*2_, which are much lower than those of CLPA and OOPDP.

In summary, in the cloud-assisted HealthIIoT system, it is crucial to perform an efficient real-time data preprocessing before outsourcing data to the cloud. PAMSD designs a new online/offline tag generation mechanism to significantly reduce the online computation costs, which is very suitable for cloud-based real-time medical sensor data.

### Comparative experiments

Detailed comparative experiments are used to evaluate performance. All experiments are performed on a Dell workstation equipped with an Intel Xeon E3-1225 v5 CPU at 3.31 GHz, 8 GB RAM and 7200RPM SATA 2 TB in Linux system (Ubuntu 16.04.2 LTS ×64 with kernel version 4.8.0). All encryption algorithms are implemented in Python environment based on the Pairing Based Cryptography (PBC) library version 0.5.14, with the MNT d159 curve with a length of 160 bits. In addition, all experimental results are the averages of 20 trials.

#### Computation costs in the tag generation

**Fig. 2:**
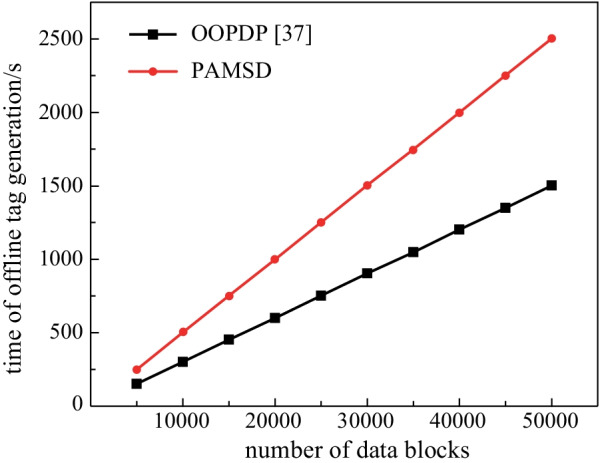
Computation costs of offline tag generation for blocks in different numbers

**Fig. 3:**
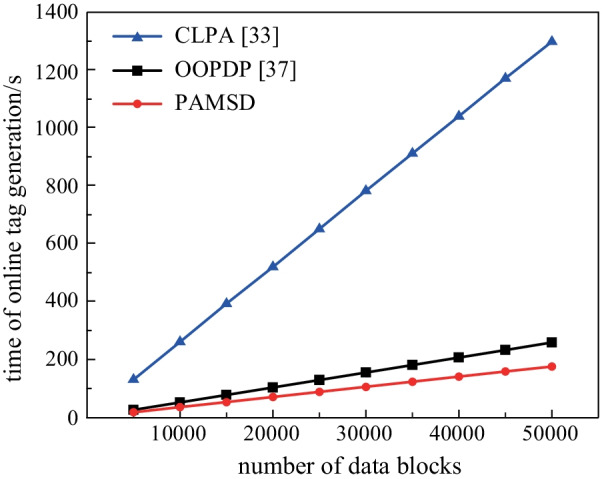
Computation costs of online tag generation for blocks in different numbers

We separately evaluate the computation costs of tag generation in online and offline phases. In the experiments, the block size is set as 4 KB, and the number of data blocks increases from 5000 to 50000 with intervals of 5000. Since the entire tag generation of CLPA is performed online, it is not included in the offline tag generation comparative experiments. Figures 2 and 3 respectively show the relationship between the offline and online tag generation time and data blocks in different numbers.

The experimental results of offline tag generation, as shown in Fig. 2, suggest that: (1) the computation costs of PAMSD and OOPDP are proportional to the number of data blocks; and (2) to preprocess the same number of data blocks, PAMSD takes more time than OOPDP.

Figure 3 shows the computation costs in the online tag generation, which shows that: (1) The time of online tag generation on all three schemes increases with the number of data blocks. (2) Under the same block number, the computation costs of PAMSD are lower than those of CLPA and OOPDP. (3) CLPA takes much more time than OOPDP and PAMSD to perform the online tag generation.

Since CLPA do not support online/offline tag generation mechanism, its computation costs of online tag generation are much higher than those of either OOPDP or PAMSD. As described in Sect. 4.3, in the offline tag generation phase, PAMSD computes the chameleon hash values with random numbers and auxiliary parameters in advance, then uses these hash values to generate offline tag pool {*σ*_*i*_}_1 ≤ *i* ≤ *k*_. Therefore, the offline computation costs of PAMSD are higher than those of OOPDP.

Considering the high real-time requirement of medical sensor data, we pay more attention to the computing overhead in the online phase that is performed by lightweight mobile terminals. The online tag generation computation costs of the presented scheme are lower than those of either CLPA or OOPDP, which is highly suitable for the cloud-assisted HealthIIoT environment. In other words, the relatively large offline computing overhead will not affect the overall performance of the system.

#### Computation costs in the verification

To evaluate the computation costs of the verification, in the comparison experiments, the block size and block number are respectively set as 4 KB and 5000, and the number of challenged blocks is increased from 300 to 460 with intervals of 20.

**Fig. 4:**
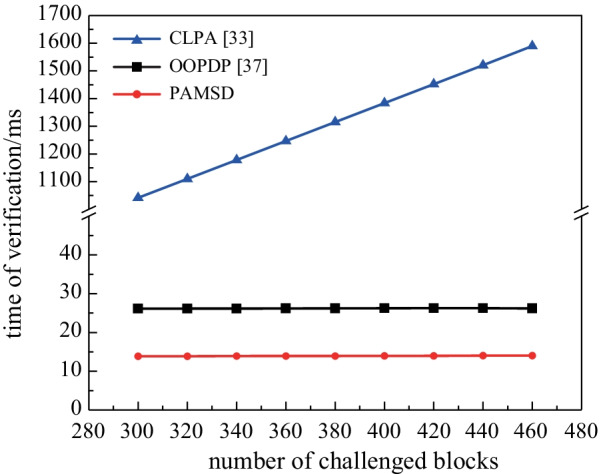
Verification time in different numbers of challenged block

Figure 4 shows the experimental result of the verification time in different numbers of challenge blocks, from which we can learn that: (1) In the three schemes, the verification time of TPA is proportional to the number of challenge blocks, but the growth rate of PAMSD and OOPDP, both of which has a much smaller initial verification time (the number of challenge blocks is equal to 300), is much lower than that of CLPA. (2) The computation costs of PAMSD in the verification phase are lower than those of CLPA and OOPDP.

Compared with CLPA, the online/offline tag generation mechanism greatly improves the verification efficiency. Meanwhile, in comparison with OOPDP, which is also based on the online/offline signature, PAMSD reduces the computation costs of the TPA in the verification phase while improving the efficiency of online tag generation. In a word, PAMSD is superior to both CLPA and OOPDP in verification performance.

## Conclusion

The cloud-assisted HealthIIoT system significantly improves healthcare services, where wireless medical sensors continuously collect real-time medical data concerning patients’ vital health parameters, and the flexible access to these cloud-based medical sensor data enables doctors to perform timely medical monitoring and diagnosis. Aiming to address data integrity issues for real-time medical sensor data in the cloud, which is crucial to the cloud-assisted HealthIIoT system, this paper presents an efficient public auditing scheme based on online/offline signature. To address the contradiction between the high real-time requirement of medical sensor data and the limited computing power of HealthIIoT devices, we design a novel online/offline tag generation algorithm. Most of the heavy computations are conducted in the offline phase before receiving medical sensor data to be outsourced, therefore, the online tag generation requires only lightweight preprocessing. Moreover, we employ the secure hash function to blind auditing proof to protect data privacy. We formally prove the security of the presented scheme, and evaluate the performance through theoretical analyses and experimental comparisons with the state-of-the-art ones. The results show that the presented scheme can significantly improve the efficiency of tag generation, while achieving better auditing performance than previous schemes.
